# Melatonergic Receptors Mediate Reduction in Ethanol Consumption in Wistar Rats

**DOI:** 10.3390/biom16040514

**Published:** 2026-03-31

**Authors:** Zahoor Ahmad Rather, Sheetal Dinkar Ullal, Muralidhara Yadiyal Baregundi, Ashok K. Shenoy

**Affiliations:** 1 Department of Pharmacology, Government Medical College, Handwara 193221, India; zahoorkmc@gmail.com; 2 Department of Pharmacology, Kasturba Medical College Mangalore, Manipal Academy of Higher Education, Manipal 576104, India; sheetal.ullal@manipal.edu; 3 Department of General Medicine, Kasturba Medical College Mangalore, Manipal Academy of Higher Education, Manipal 576104, India; yadiyal.m@manipal.edu

**Keywords:** melatonergic receptors, Wistar rats, ethanol consumption, alcohol dependence

## Abstract

**Introduction:** Ethanol consumption is a major global health concern associated with many disorders. Melatonin, a circadian neurohormone, also modulates dopamine signaling and drug-seeking behaviors central to addiction. **Objective:** To investigate the role of melatonergic receptors in reducing ethanol consumption and explore melatonin’s therapeutic potential to overcome ethanol dependence in rats. **Method:** Male Wistar rats were acclimatized for 5 days, then divided into two groups: ethanol-naive (*n* = 6) and ethanol-exposed (*n* = 36). The ethanol group was given 10% ethanol (7 days), followed by limited access (27 days). On day 40, the ethanol group was divided into 6 sub-groups, receiving various treatments, including melatonin and receptor blockers (10 days). Ethanol and water consumption were measured daily. On day 49, the rats were sacrificed, and dopamine levels in the nucleus accumbens were quantified using an ELISA. **Results:** Both melatonin doses and naltrexone significantly reduced ethanol consumption (*p* < 0.05) compared to their pretreatment levels. Ethanol reduction was greater in the melatonin-treated groups than the control group (*p* = 0.004, *p* = 0.007). However, the melatonin’s efficacy was blocked when coadministered with receptor antagonists, showing no significant ethanol reduction vs. control (*p* = 0.075 and *p* = 0.08). **Conclusions:** These findings suggest that melatonin reduces ethanol intake via specific receptor pathways, supporting its potential use in treating alcohol dependence.

## 1. Introduction

The use of ethanol by humans dates back before written history and is thought to confer adaptive evolutionary benefits [1]. In modern life, alcohol is one of the most widely consumed psychoactive drugs globally. Hazardous drinking, marked by high intake and frequent consumption, is associated with both short- and long-term morbidity [2]. Lifetime alcohol consumption exceeds 80% among adults in most high-income nations, whereas prevalence in low- and middle-income countries is more heterogeneous. At least annual consumption is reported by the majority of adults in Europe (59.9%), the Americas (54.1%), and the Western Pacific region (53.8%). Globally, an estimated 2.3 billion adults consume alcohol at least once yearly [3]. Substance abuse poses a serious worldwide health challenge with far-reaching effects on individual well-being and societal functioning. Drugs of abuse modulate neural function through complex mechanisms involving molecular signaling, neuronal and glial interactions, and alterations in neural circuitry. Such changes underlie the physiological and behavioral adaptations that impact core domains including reward, motivation, cognitive processes, stress regulation, and decision-making capacity [4,5]. The core regions of the mesolimbic dopaminergic circuit, including the ventral tegmental area (VTA), nucleus accumbens (NAc), and amygdala, play pivotal roles in governing reward-related signaling and motivational behaviors [6].

The primary objectives of addiction management are: (a) attenuation of substance use and associated craving; (b) enhancing overall health and functional outcomes; and (c) mitigating the chances of adverse complications and relapse [7].

As ethanol dependence affects millions of individuals throughout the world, effective management of the condition is essential. Though there has been some progress in the pharmacotherapy of this disorder, these therapies may not be effective for all individuals. Existing treatment strategies remain insufficient, with relapse rates demonstrating significant variability across different substances of abuse [8]. Drugs like naltrexone, acamprosate and disulfiram are used in the management of ethanol dependence. However, maintaining abstinence after withdrawal and preventing relapse is still a major challenge. In addition, these drugs have certain limitations. The efficacy of naltrexone may be limited in certain patients, potentially due to genetic polymorphisms in the µ-opioid receptor gene [9]. Disulfiram and naltrexone are associated with the rise in liver enzymes and a higher tendency of developing hepatotoxicity in high-risk patients with already deranged liver enzymes [10]. The global burden of ethanol-associated liver disease is considered one of the most frequent causes of death worldwide [11]. Although ethanol itself is associated with liver disease, exacerbation or worsening of the condition may occur with the use of these drugs, especially when they are used for a prolonged duration [12].

The complexity of genetic and environmental factors involved in the development of ethanol dependence demands the search for newer pharmacological interventions that are effective across all patient populations [13]. Thus, exploring additional therapeutic approaches and continuing research to discover new drugs are necessities in this area.

Emerging evidence highlights melatonin’s potential to modulate key neurobiological substrates of addiction, with preclinical studies demonstrating its efficacy in attenuating drug-seeking behavior, preventing opiate withdrawal and relapse, and reducing behavioral sensitization [14,15,16]. All the agents known to cause dependence, such as ethanol, opioids, cocaine, amphetamine, cannabinoids, and nicotine, have reinforcing properties that are known to enhance neuronal activity in specific brain regions. These agents elevate the extracellular dopamine neurotransmitter levels in the nucleus accumbens [17].

Melatonin also has an inhibitory effect on the NMDA (N-methyl-D-aspartate) receptor, thereby suppressing glutaminergic excitotoxicity and it has been shown to have a prominent effect on the opioid system as well [18]. The morphine-induced rewarding effect has been shown to be reversed by melatonin by the activation of the melatonergic MT2 receptor subtype in the CNS [19]. The ability of melatonin to reverse morphine dependence and tolerance may involve the suppression of nitric oxide synthase activity [20]. And with all of this evidence, we decided to conduct this study to explore the possibility of the use of melatonin in ethanol dependence treatment.

## 2. Materials and Methods

This preclinical study investigated the effects of melatonin and its receptors on ethanol consumption and dopamine levels in Wistar rats. It involved the controlled manipulation of variables (ethanol consumption and drug administration) to assess outcomes, using male Wistar rats divided into ethanol-naive and ethanol-exposed groups. The ethanol group was given 10% *v*/*v* ethanol solution, followed by limited access for six hours, then treated with drugs (including melatonin and receptor blockers) administered for 10 days. Ethanol and water consumption were measured, and dopamine levels in the nucleus accumbens were assessed after sacrificing the animals on day 49.

### 2.1. Animals

Before initiating the experimental protocol, the male Wistar rats (7 weeks; 200–250 g) underwent a 5-day acclimatization period in the animal facility, where they were kept individually in standard polycarbonate cages (29 cm × 22 cm × 14 cm) with paddy husk bedding under controlled temperature (21 ± 1 °C) and photoperiod (12:12 L:D). Food pellets (Agro Industries Ltd., Pune, India) containing 0.2–0.5% tryptophan to enhance the melatonin synthesis, were available to animals on an ad libitum basis. The measured volume of 100 mL of water and/or 10% *v*/*v* ethanol was filled in a plastic bottle of 125 mL capacity and available for the rats for a specific period of time, as explained in the experimental procedure. Appropriate measures were implemented to ensure that no leakage occurred from the bottles containing the drinking solution. All experimental procedures were strictly scheduled between 09:00 and 16:00 h to mitigate any variabilities related to circadian rhythm. The whole study was executed in full compliance with the ethical and regulatory standards stipulated by the Committee for the Purpose of Control and Supervision of Experiments on Animals [21]. All possible measures were implemented to ensure the well-being and reduce discomfort to the experimental animals. Institutional Animal Ethical Committee approval was obtained before the commencement of study.

### 2.2. Drugs and Drinking Solutions

The ethanol-naïve group received tap water as their sole drinking solution throughout the study, whereas the ethanol-exposed group was provided with tap water following the conclusion of the limited ethanol access period. A 10% (*v*/*v*) ethanol drinking solution was formulated by diluting absolute anhydrous ethanol (99.9%; Changshu Yangyuan Chemicals, Changshu, China) with tap water. Melatonin (M5250; Sigma-Aldrich, St. Louis, MO, USA) was prepared by using distilled water and injected through the intraperitoneal route, with doses of 25 mg/kg and 50 mg/kg selected in accordance with the previously published literature [22]. Luzindole (L2407; Sigma-Aldrich, St. Louis, MO, USA) was dissolved in distilled water and administered through the intraperitoneal route (1 mg/kg). Prazosin (P7791; Sigma-Aldrich, St. Louis, MO, USA) was dissolved in distilled water and administered through the intraperitoneal route (1 mg/kg). Naltrexone (N3136; procured from Sigma-Aldrich, St. Louis, MO, USA) pure powder was dissolved in distilled water as a standard drug and administered at a dose of 1 mg/kg intraperitoneally [23]. Distilled water was used as a vehicle.

### 2.3. Experimental Procedure

**Figure 1:**
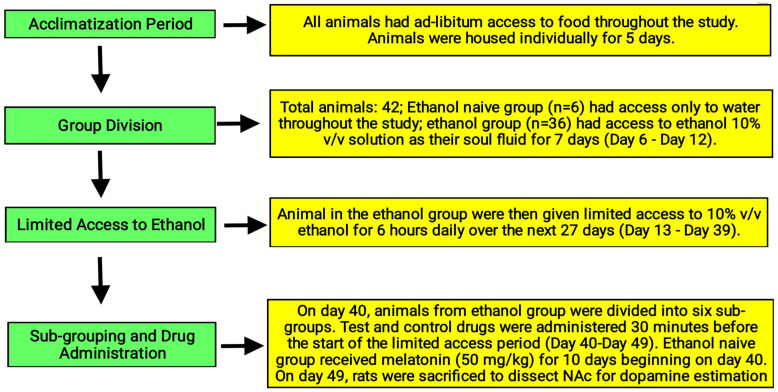
Experimental design and treatment timeline of the ethanol consumption model in Wistar rats.

**Figure 2:**
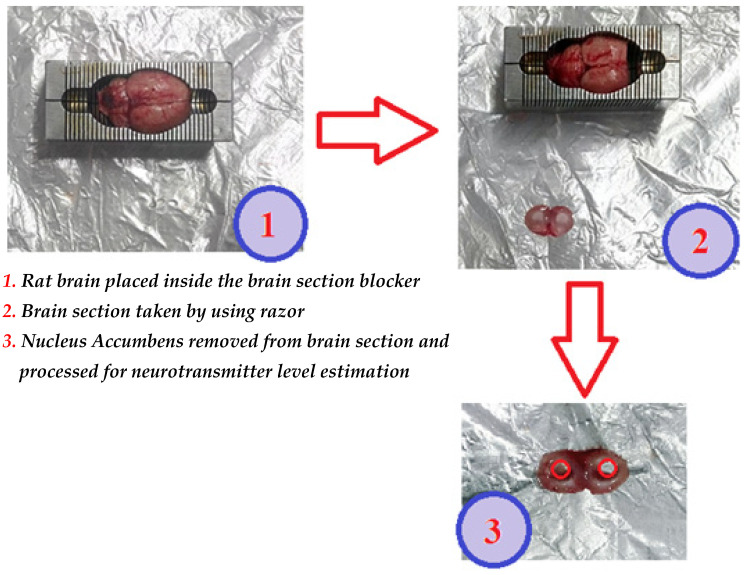
Rat brain sectioning and extraction of nucleus accumbens. Paxinos, G; Watson, C. The Rat Brain in Stereotaxix Coordinates—The New Coronal Set; Elsevier: New York, NY, USA, 2004 [24].

All animals had ad libitum access to food throughout study. After the acclimatization period (i.e., for 5 days), individually caged animals (n = 42) were divided into two main groups. Out of these groups, one group (ethanol-naive group; n = 6) had access to only water throughout study without being given the ethanol drinking solution. The other group (ethanol group; n = 36) had access to ethanol 10% *v*/*v* solution as their sole fluid for 7 days (from days 6 to 12), i.e., they were forced to drink ethanol as the only solution during this period. Following this, the animals from the ethanol group had limited access to ethanol 10% for 6 h (10:00 h to 16:00 h ) for the next 27 days (from days 13 to 39); for the remaining 18 h the animals had access to water. Ethanol consumption by rats was measured at the end of the limited ethanol session daily and the same procedure was followed to measure the water consumption by rats on daily basis. On day 40, the animals from the ethanol group were divided into six sub-groups (named as group-1 to group-6, respectively; n = 6 rats in each group) randomly by matching for ethanol consumption [experimental design shown in Table 1]. Test and control drugs were administered 30 min before the beginning of the limited access period for 10 days (i.e., from days 40 to 49) (Figure 1). For the ethanol-naïve group, water consumption was recorded at the same time points that were used in the ethanol-exposed groups. This group received melatonin at a dose of 50 mg/kg beginning on day 40 for 10 days (i.e., the same time point at which different drugs were administered to the ethanol-exposed rats depending on their allocated groups). The drug administration schedule is described in Table 2. The mean ethanol consumption of two days preceding the initiation of drug administration was taken as baseline values. On day 49, the rats were sacrificed and the nucleus accumbens was dissected out and processed for the estimation of dopamine levels as shown in Figure 2.

### 2.4. Dissection of Discrete Region of the Brain

All the samples of the nucleus accumbens were collected between 16:00 h. and 17:00 h. in order to avoid circadian rhythm-induced variation. Rats were sacrificed by cervical dislocation followed by immediate decapitation to remove the brain. To expose the brain, the tip of the curved scissors was inserted into the foramen magnum and a single lateral cut was made into the skull, extending forward on the left and right side. With the help of a bone cutter, the dorsal portion of the cranium was peeled off and by means of blunt forceps, the brain was put onto an ice-cold glass plate, leaving behind the olfactory bulbs. The nucleus accumbens was quickly dissected out by using a brain sectioning blocker and by following the dissection guidelines given in the brain atlas by Paxinos et al. [24]. The brain sections were kept on the chilled aluminum foil placed on crushed ice, as shown in Figure 2. The brain sample was then frozen and stored immediately at −70 °C or below until subsequent homogenization followed by neurotransmitter level estimation.

Tissue preparation: On the day of the assay, the wet weight of the brain tissue was recorded, placed in an ice-cooled 0.1 N HCl and 1 mmol EDTA solution (1 mL/50 mg wet weight sample), and homogenized. Following centrifugation at 15,000× *g* for 15 min at 4 °C, the supernatant was collected for enzyme-linked immunosorbent assay (ELISA) analysis.

### 2.5. ELISA Analysis of Neurotransmitter Levels of Dopamine in the Nucleus Accumbens

Principle of the assay: The quantification of dopamine levels in the nucleus accumbens of Wistar rats was performed using an ELISA based on the sandwich principle.

Assay procedure: Dopamine was quantified using a commercially available Dopamine ELISA kit (Immuno-Biological Laboratories, Hamburg, Germany) according to the manufacturer’s protocol.

Calculation of dopamine levels: The level of neurotransmitters in each sample was determined using the standard curve. The concentrations of dopamine in the kit controls and the samples in ng/mL could be read directly from the corresponding standard curve.

In the groups (group-1 to group-6), the animals had limited access to ethanol 10% for 6 h (10:00 h–16:00 h) and for the rest of the time (next 18 h) they had access to only water. The control group (group-1) receiving distilled water will serve as a negative control and the control group (group-4) receiving standard drug naltrexone will serve as a positive control. In group 7, the animals were ethanol-naïve and after receiving the melatonin treatment, the rats were exposed only to water on an ad libitum basis, but not to ethanol. This group was included, just to rule out the possibility of the sedative effect of melatonin on ethanol drinking among rats (Table 2).

### 2.6. Statistical Analysis

The data was analyzed by using SPSS version 16. We used non-parametric tests (Wilcoxon, Mann–Whitney) when the data violated normality assumptions. Parametric (one-way ANOVA) was used where the distribution was normal and variances were homogeneous. For non-parametric data, the Wilcoxon signed-rank and Mann–Whitney U tests were used to compare the differences in the consumption of drinking solution within the groups as well as in between the different groups at different time points. Group comparison was carried out using one-way ANOVA, after which a post hoc test (Tukey’s) was run on multiple comparisons. A *p*-value of less than 0.05 was taken as significant.

## 3. Result

**Figure 3:**
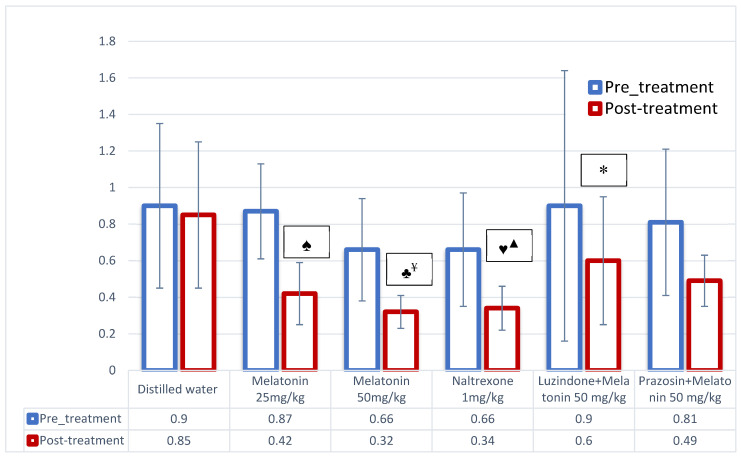
Pretreatment and post-treatment as well as intergroup comparison of ethanol in FEC model. Values are expressed as mean ± SD; Wilcoxon signed-rank test: within the group comparison. Mann–Whitney U test: for intergroup comparison. ♠ *p* = 0.028; post-treatment ethanol consumption of melatonin 25 mg/kg (0.42 ± 0.17) vs. its pretreatment levels (0.87 ± 0.26). ♣ *p* = 0.018; post-treatment ethanol consumption of melatonin 50 mg/kg (0.32 ± 0.09) vs. its pretreatment levels (0.66 ± 0.28). ♥ *p* = 0.018; post-treatment ethanol consumption of naltrexone 1 mg/kg (0.34 ± 0.12) vs. its pretreatment levels (0.66 ± 0.31). ^¥^ *p* = 0.004; post-treatment ethanol consumption of melatonin 50 mg/kg (0.32 ± 0.09) vs. distilled water (0.85 ± 0.40). ^▲^ *p* = 0.007; post-treatment ethanol consumption of naltrexone 1 mg/kg (0.34 ± 0.12) vs. distilled water (0.85 ± 0.40). * *p* = 0.032; post-treatment ethanol consumption of luzindole + melatonin 50 mg/kg (0.60 ± 0.35) vs. melatonin 50 mg/kg (0.32 ± 0.09).

In this ethanol consumption model, melatonin 25 mg/kg-, melatonin 50 mg/kg- and naltrexone 1 mg/kg-treated groups showed a statistically significant reduction in ethanol consumption compared to their pretreatment ethanol levels (0.42 ± 0.17 g/kg vs. 0.87 ± 0.26 g/kg; 0.32 ± 0.09 g/kg vs. 0.66 ± 0.28 g/kg; 0.34 ± 0.12 g/kg vs. 0.66 ± 0.31 g/kg, respectively) (*p* = 0.028, *p* = 0.018 and *p* = 0.018, respectively). However, the effect of melatonin in reducing the ethanol consumption was diminished in the luzindole (*p* = 0.032)-treated groups, which shows that luzindole blocked the effect of melatonin on ethanol consumption (Figure 3).

There was also a significant difference between the melatonin 50 mg/kg (0.32 ± 0.09 g/kg)- and naltrexone 1 mg/kg (0.34 ± 0.12 g/kg)-treated groups when compared with the distilled water group (0.85 ± 0.40 g/kg) (*p* = 0.004 and *p* = 0.007, respectively). None of the other groups showed any significant difference compared to the distilled water group (Figure 3).

The difference in post-treatment ethanol consumption is significant between melatonin (50 mg/kg)- and luzindole + melatonin-treated groups, whereas there is no significant difference in ethanol consumption between melatonin (50 mg/kg)- and prazosin + melatonin-treated groups (Figure 3). Hence, luzindole appears to be more efficacious than prazosin in blocking the effect of melatonin on ethanol consumption.

None of these drugs had any influence on water consumption as there was no significant difference seen between the pretreatment and post-treatment water consumption (by using the Wilcoxon signed-rank test) in all the groups. The water consumption was comparable between the groups as the intergroup comparison did not show any statistical significance (by the Mann–Whitney U test) (Table 3).

The water-fed group when treated with melatonin 50 mg/kg did not show any significant difference in water consumption when compared with pretreatment baseline water consumption. This group was included, just to rule out the possibility of the sedative effect of melatonin on ethanol drinking among rats (Table 4).

There was a gradual increase in body weight seen in post-treatment in every drug-treated group. This increase in body weight did not show any statistically significant difference among any drug-treated group (Table 5).

### Neurotransmitter Levels

**Figure 4:**
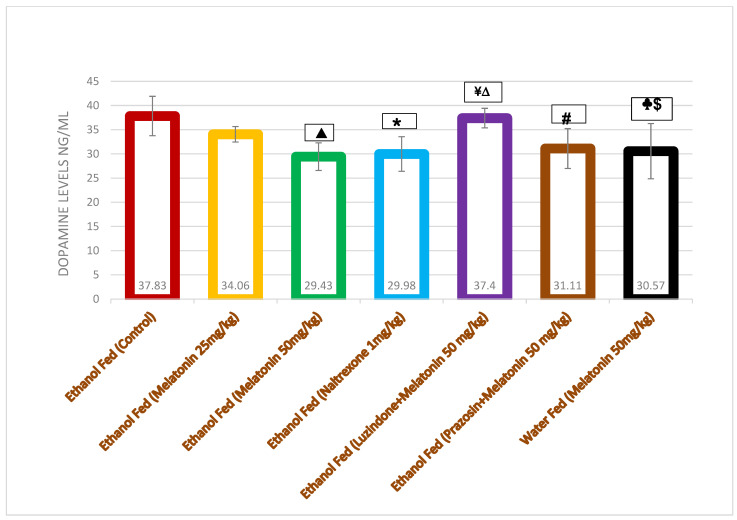
Influence of drugs on dopamine levels in nucleus accumbens in Wistar rats. Values expressed in mean ± SD; ONE-WAY ANOVA followed by Tukey’s (post hoc). ^▲^ *p* = 0.006; ethanol-fed melatonin 50 mg/kg (29.43 ± 2.86) vs. ethanol-fed control (37.83 ± 4.07). * *p* = 0. 011; ethanol-fed naltrexone 1 mg/kg vs. ethanol-fed control. ^¥^ *p* = 0.01; ethanol-fed luzindole + melatonin 50 mg/kg (37.40 ± 2.02) vs. ethanol-fed melatonin 50 mg/kg (29.43 ± 2.86). ^∆^ *p* = 0.019; ethanol-fed luzindole + melatonin 50 mg/kg (37.40 ± 2.02) vs. ethanol-fed naltrexone 1 mg/kg (29.98 ± 3.58). # *p* = 0.044; ethanol-fed prazosin + melatonin 50 mg/kg (31.11 ± 4.11) vs. ethanol-fed (control). ^♣^ *p* = 0.023; water-fed melatonin 50 mg/kg (30.57 ± 5.70) vs. ethanol-fed (control). ^$^ *p* = 0.038; water-fed melatonin 50 mg/kg (30.57 ± 5.70) vs. ethanol-fed (luzindole + melatonin 50 mg/kg). dF: between groups = 6, within groups = 30; F = 4.747.

The dopamine levels were higher in the ethanol-consuming control animals compared with the drug-treated ethanol-exposed groups. Melatonin 50 mg/kg as well as naltrexone 1 mg/kg reduced the dopamine levels significantly (29.43 ± 2.86 ng/mL, 29.98 ± 3.58 ng/mL; *p* = 0.006 and 0.011, respectively) when compared with the distilled water (negative control) group (37.83 ± 4.07 ng/mL). The effect of melatonin was dose dependent. There was a statistically significant augmented release of dopamine seen in the luzindole-treated group (37.40 ± 2.02 ng/mL) compared with melatonin 50 mg/kg (29.43 ± 2.86 ng/mL), naltrexone 1 mg/kg (29.98 ± 3.58 ng/mL) and the ethanol-naïve group treated with melatonin 50 mg/kg (30.57 ± 5.70) (*p* = 0.01; 0.019; 0.038, respectively). However, the prazosin-treated group did not show any statistical significance when compared with melatonin 50 mg/kg, naltrexone 1 mg/kg and the ethanol-naïve group treated with melatonin 50 mg/kg. These observations indicate that the actions of melatonin might occur via the activation of central MT_1_ and MT_2_ melatonergic receptors (blocked by luzindole) in the nucleus accumbens to reduce the ethanol consumption and thereby reduce dopamine levels, whereas the MT_3_ receptor (blocked by prazosin) may not be involved in such an activity (Figure 4).

## 4. Discussion

In the forced ethanol consumption model, animals were given access to an ethanol solution as their sole source of fluid for a restricted period. During this time, they were compelled to consume ethanol, which facilitates achieving pharmacologically relevant levels of ethanol in rodents [25]. The findings from our study using this model demonstrated that both melatonin as well as naltrexone significantly diminished ethanol intake in high-ethanol-consuming rats. However, ethanol consumption was not significantly altered when the animals were pre-treated with melatonin receptor antagonists such as luzindole and prazosin before receiving melatonin (Figure 3). These observations suggest that the effect of melatonin on reducing ethanol consumption may be mediated through its action on melatonergic receptors, and that this effect appears to be blocked by luzindole (MT_1_ and MT_2_ receptor blocker). Based on these results, we can infer that the influence of melatonin on ethanol consumption may be mediated through the involvement of MT_1_ and MT_2_ melatonergic receptors. Furthermore, our data indicate that luzindole exerted a stronger blocking effect on melatonin’s action compared to prazosin, as a significant difference in ethanol intake was found only between the luzindole + melatonin group and the melatonin (50 mg/kg) group. The prazosin-treated group did not display any difference in ethanol intake compared to the melatonin 50 mg/kg-treated group (Figure 3), and there was no significant difference when compared to its pretreatment baseline values.

We also included a group of animals with access to water as the sole drinking solution (i.e., the water-fed group) during the restricted period instead of ethanol. These animals received melatonin (at a higher dose at the same time point at which different drugs were administered to the ethanol-exposed rats depending on their allocated groups) and the water consumption was recorded for this group at the same time points that were used in the ethanol-exposed groups. The results showed that the water-fed group did not show any significant difference in water consumption when compared with the pretreatment baseline water consumption (Table 4). These findings suggest that melatonin did not have any effect on the normal drinking behavior of the animals and thus rules out the possibility of the sedative effect of melatonin affecting ethanol consumption.

Dopamine levels in the NAc were significantly higher in the rats receiving distilled water which had high ethanol consumption compared to the levels of dopamine in the melatonin- and naltrexone-treated groups (Figure 4). Thus, the groups which showed an increase in ethanol consumption also had a relatively elevated dopamine level in their NAc and vice versa. This demonstrates a correlation between ethanol consumption and dopamine levels in the NAc.

The effect of melatonin on ethanol consumption and dopamine level could be through the involvement of melatonin receptors (MT_1_ and MT_2_) present in the NAc, which was reinforced by administering melatonin receptor antagonists like luzindole (MT_1_ and MT_2_ receptor blocker). The suppressive effect of melatonin on ethanol consumption was not seen in animals treated with luzindole + melatonin, i.e., there was a statistically significant increase in ethanol intake which augmented the release of dopamine in the luzindole-treated group (Figure 3 and Figure 4). However, the animals treated with prazosin did not show any statistically significant difference in ethanol consumption and dopamine level when compared with melatonin 50 mg/kg- or naltrexone-treated groups (Figure 3 and Figure 4). This shows that melatonin acts through MT_1_ and MT_2_ receptors (blocked by luzindole) to reduce ethanol consumption and thereby dopamine levels, whereas the MT_3_ receptor (blocked by prazosin) may not be involved in such an effect.

Melatonin in the brain is known to regulate various physiological and neuroendocrine functions. Chronic alcohol consumption not only reduces melatonin levels but also delays the pineal melatonin production [26]. Heavy alcohol use is associated with a decrease in melatonin level and may contribute to alcohol-induced sleep disturbance [27].

The potential efficacy of melatonin has been demonstrated in several types of drug dependence in preclinical studies, suggesting that melatonin has some reversal effects on different drugs of abuse and can be useful in some aspects of drug dependence [14]. Treatment with melatonin effectively counteracted morphine-induced tolerance and dependence as well as mitigated withdrawal-related behaviors in mice [20,28,29,30,31]. Reversal of the expression of the morphine-induced rewarding effect by melatonin may be mediated by the activation of the melatonin MT_2_ receptor subtype within the central nervous system [19].

Studies have demonstrated that physiological doses of melatonin diminish craving in heavy smokers undergoing acute nicotine withdrawal [32]. Moreover, melatonin plays a role in cocaine-induced reward and has been linked to reduced long-term benzodiazepine consumption among insomnia patients [33,34]. Therapeutic modulation of the melatonergic system with melatonin in alcohol-dependent patients, particularly those presenting with comorbid depression, may restore normal sleep pattern, attenuate alcohol craving, and consequently reduce relapse risk [35].

Dopamine signaling plays a critical role in mediating the reinforcing effects of dependence-inducing substances, including ethanol [17]. The dopaminergic mesolimbic system is integral to motivational and reinforcement processes underlying behavior. Ethanol enhances dopaminergic transmission within this pathway and elevates the firing rate of dopaminergic neurons, thereby promoting dopamine release. The transient dopamine surge induced by dependence-producing agents in these regions contributes significantly to their reinforcing properties. Consumption of sweet fluids in rodents elicits a transient dopamine surge, potentially mediating the reinforcement of sugar water drinking [36], with similar dopaminergic responses reported in humans [37].

Genetic studies indicate that single nucleotide polymorphisms (SNPs) in dopaminergic pathway genes are associated with an elevated risk of ethanol dependence [38]. Dopamine release at VTA has a clear role in the initiation of ethanol consumption in the nucleus accumbens [39,40,41].

Compelling evidence for dopamine’s involvement in ethanol reward is provided by findings that rats voluntarily self-administering ethanol show an increased dopamine release in the nucleus accumbens [42]. Chronic ethanol abuse often necessitates the consumption of larger ethanol quantities to trigger dopamine release and elicit its rewarding effects. During ethanol withdrawal, dopamine release diminishes, leading to reduced neuronal firing, which manifests as dysphoria, malaise, and depression [43]. Ethanol modulates mesolimbic dopamine system activity through its action on opioid receptors within the VTA and NAc [17], and studies indicate that this modulation occurs via direct or indirect interactions with opioid receptors [44].

Melatonin receptors are found in several locations that receive dopaminergic innervation, such as the prefrontal cortex, striatum, NAc and amygdala in the mammalian brain [45,46]. The inhibition of dopamine release by melatonin has been demonstrated in sites such as the hypothalamus, hippocampus, medulla-pons, and retina [47,48]. Studies have proven the anti-dopaminergic activities of melatonin in the striatum. From various studies, it has been demonstrated that melatonin in physiological doses generally produces an anti-dopaminergic effect on the brain [47], while high doses of melatonin have no effect on dopaminergic neurons [49]. The melatonin doses in our study, 25 mg/kg and 50 mg/kg, were much higher than the physiological doses, and at these doses, we too did not see the stimulating effect of melatonin on dopamine release.

Several studies have demonstrated the efficacy of naltrexone in ethanol dependence with progress in maintaining abstinence and suppression of heavy drinking [44,50,51,52]. It produces its effects through interaction with dopamine and the endogenous opioid systems [53]. The inhibitory effect of naltrexone on GABAergic neurons in the ventral midbrain may affect the dopamine reward pathway in ethanol dependence [54].

The findings of the present study suggest that melatonin plays a significant role in modulating ethanol consumption, aligning with previous research demonstrating its ability to reduce alcohol-seeking behavior and alleviating withdrawal symptoms in various animal models of substance dependence [14,20,35]. The observed reduction in ethanol intake following melatonin administration is consistent with earlier studies showing that melatonin supplementation decreases alcohol consumption and influences dopaminergic pathways implicated in addiction mechanisms [35]. Moreover, our data demonstrate that the pharmacological effects of melatonin were completely abolished by luzindole, a non-selective MT_1_/MT_2_ receptor antagonist, further supporting the hypothesis that melatonin’s action is mediated through its central melatonergic receptors [35]. These observations reinforce the notion that melatonergic receptors—particularly MT_1_ and MT_2_—play a key role in regulating ethanol intake and associated dopaminergic signaling within the nucleus accumbens.

Our observations regarding melatonin in reducing the ethanol consumption in Wistar rats strongly suggest the possible inhibitory role of melatonin in ethanol consumption. The use of a melatonin antagonist, i.e., luzindole, attenuated the effect of melatonin on ethanol consumption, suggesting the association of MT_1_ and MT_2_ receptors in the above observations. Further ethanol consumption increased dopamine levels in the nucleus accumbens, suggesting that the principal neurotransmitter involved in ethanol dependence was dopamine. A decrease in dopamine in NAc following melatonin and naltrexone treatments strongly suggests that the neurocircuitry of melatonin action could involve the nucleus accumbens–dopaminergic pathway.

Pharmacological agents reducing ethanol intake may induce compensatory increases in water intake, but such compensation was not observed in our study. This may suggest a specific effect of melatonin on ethanol-directed motivation rather than general fluid regulation.

Our study demonstrates that melatonin effectively reduces ethanol consumption in rats, suggesting its potential as a therapeutic agent for alcohol dependence. Melatonin’s effects were mediated through melatonin receptors, particularly MT_1_ and MT_2_, as indicated by the attenuation of its action when the receptor blocker luzindole was administered. However, Froehlich JC et al. have shown that prazosin reduces alcohol drinking throughout prolonged treatment and blocks the initiation of drinking in rats [55].

Therefore, our study suggests that melatonin could be a candidate molecule in the treatment of ethanol dependence. Further studies are required to assess the efficacy in clinical trials.

There are several limitations in this study. First, only male Wistar rats were applied; there was a lack of assessment regarding the effects of sex differences in the melatonin metabolism and addiction biology. Second, investigations were restricted to short-term administration and the effects of melatonin were not tested chronically. Third, plasma (or brain) melatonin concentrations were not assessed. Fourth, a melatonin-free ethanol-naive group, if included, would provide an additional baseline for dopamine levels. Fifth, we could have introduced groups receiving luzindole or prazosin alone. The comparison of ethanol intake and dopamine levels in NAc between luzindole + melatonin and luzindole alone would have confirmed the role of melatonin receptors in reducing ethanol consumption. Lastly, there was no response to genetic and behavioral variability that could affect treatment response. Researchers ought to investigate these factors in the future to augment the relevance of translations. In future translational studies, the inter-individual variability of melatonin efficacy is to be considered. Patients with alcohol use disorder whose sleep is disrupted or serotonergic dysregulated may respond better to melatonin therapy in alcohol use disorder. It shows the importance of individual treatment and biomarker-directed clinical trials.

## 5. Conclusions

The present study demonstrates that melatonin significantly reduces ethanol consumption in high-ethanol-consuming rats, indicating its potential role as a therapeutic agent in alcohol dependence. The attenuation of melatonin’s effect by luzindole, but not by prazosin, suggests that its action is primarily mediated through MT_1_ and MT_2_ melatonergic receptors, while MT3 receptors appear to have minimal involvement.

A positive correlation between ethanol intake and dopamine levels in the nucleus accumbens was observed, with melatonin and naltrexone reducing both ethanol consumption and dopaminergic activity, highlighting the role of the mesolimbic dopamine pathway. The absence of changes in water intake in the control group confirms that melatonin does not affect general fluid consumption, thereby ruling out nonspecific effects such as sedation.

Overall, these findings support the involvement of melatonergic modulation of dopaminergic neurocircuitry in ethanol dependence and suggest that melatonin could be a promising candidate for future clinical evaluation in alcohol use disorders.

## Figures and Tables

**Table 1:** Experimental design for individual rat regimen (in ethanol group).

Days 1–5	Adjustment period in individual cages.
Days 6–12	Rats were forced to drink ethanol 10% (ethanol-fed group) as sole fluid (except water-fed group) throughout the day with ad libitum access to food.
Days 13–39	Rats were exposed to limited period (6 h in 24 h) access to ethanol 10% (except water-fed group).
Days 40–49	From ethanol-fed group, rats were allocated into six groups on day 40 and received drugs (according to the groups allocated) 30 min before limited access period to 10% ethanol. Water-fed group received melatonin at higher dose as mentioned in Table 2.
Days 49	Rats were sacrificed and nucleus accumbens was dissected out and processed for the dopamine estimation.

**Table 2:** Treatment schedule for forced ethanol consumption model.

Groups	Drug (Route-Intraperitoneal, Dose) ×10 Days
1. Control group (negative control)	Vehicle (distilled water) (10 mL/kg)
2. Test drug (lower dose)	Melatonin (25 mg/kg)
3. Test drug (higher dose)	Melatonin (50 mg/kg)
4. Standard drug (positive control)	Naltrexone (1 mg/kg)
5. Prazosin + melatonin	Prazosin (1 mg/kg) + melatonin(50 mg/kg)
6. Luzindole + melatonin	Luzindole (1 mg/kg) + melatonin (50 mg/kg)
7. Melatonin (water-fed group)	Melatonin (50 mg/kg)

**Table 3:** Effect of drugs on water intake in forced ethanol consumption model.

Drugs	Water Consumption (mL)		Z Value	*p* Value
	Pretreatment	Post-Treatment		
Distilled water	25.06 ± 1.56	25.95 ± 3.61	−0.94	0.34
Melatonin 25 mg/kg	23.82 ± 2.51	23.97 ± 3.01	−0.73	0.46
Melatonin 50 mg/kg	25.55 ± 4.50	24.48 ± 4.92	−1.86	0.06
Naltrexone 1 mg/kg	26.53 ± 2.00	24.94 ± 3.55	−0.85	0.40
Luzindole + melatonin 50 mg/kg	23.69 ± 2.40	23.75 ± 1.91	−0.94	0.34
Prazosin + melatonin 50 mg/kg	27.68 ± 7.16	27.78 ± 6.40	−0.52	0.60

Values are expressed as mean ± SD; Wilcoxon signed-rank test.

**Table 4:** Effect of melatonin on water intake in water-fed group of forced ethanol consumption model.

Drug	Water Consumption (mL)		*t* Value	*p* Value
	Pretreatment	Post-Treatment		
Melatonin 50 mg/kg (water-fed group)	9.64 ± 3.01	9.80 ± 2.54	−0.21	0.85

Values are expressed as mean ± SD; Student’s ‘*t*’ test.

**Table 5:** Effect of drugs on body weight in forced ethanol consumption model.

Drugs	Body Weight (Grams)	
	Pretreatment	Post-Treatment
Distilled water	309.16 ± 24.33	313.50 ± 25.49
Melatonin 25 mg/kg	316.83 ± 10.94	324.66 ± 11.43
Melatonin 50 mg/kg	344.28 ± 34.64	362.14 ± 38.77
Naltrexone 1 mg/kg	341.14 ± 39.31	353.28 ± 47.49
Luzindole + melatonin 50 mg/kg	333.33 ± 26.24	343.16 ± 25.75
Prazosin + melatonin 50 mg/kg	339.00 ± 37.41	344.00 ± 38.18
Water-fed group (melatonin 50 mg/kg)	355.67 ± 29.16	373.67 ± 33.33

Values are expressed as mean ± SD; ONE-WAY ANOVA followed by Tukey’s post hoc test.

## Data Availability

The original contributions presented in this study are included in the article. Further inquiries can be directed to the corresponding author.
